# The Subjective Well-Being of Elderly Migrants in Dongguan: The Role of Residential Environment

**DOI:** 10.3390/tropicalmed7080199

**Published:** 2022-08-21

**Authors:** Yuxi Liu, Li Jia, Junhui Xiao, Qin Chen, Qihui Gan, Jie Huang, Xianglei Zhu, Chichen Zhang, Chonghua Wan

**Affiliations:** 1 School of Humanities and Management, Guangdong Medical University, Dongguan 523808, China; 2 Institute of Health Law and Policy, Guangdong Medical University, Dongguan 523808, China; 3 School of Health Management, Southern Medical University, Guangzhou 510515, China; 4 Institute of Health Management, Southern Medical University, Guangzhou 510515, China; 5 Department of Health Management, Nanfang Hospital, Guangzhou 510515, China; 6 Research Center for Quality of Life and Applied Psychology, Guangdong Medical University, Dongguan 523808, China

**Keywords:** subjective well-being, residential environment, elderly migrants, China

## Abstract

To examine the association between community and individual-level residential environment in relation to subjective well-being (SWB) amongst 470 elderly migrants in China, this community-based survey was conducted. The manner and extent to which the SWB of these elderly migrants is influenced by their residential environment was the main area of focus. The Scale of Happiness of the Memorial University of Newfoundland was used to assess SWB. SWB was found to be associated significantly with environmental factors such as social cohesion, closeness to the nearest facility of recreation, the density of recreation facilities, financial facilities, and health facilities. The health facility density (B = 0.026, *p* < 0.001) and recreation facility density (B = 0.032, *p* < 0.001) had positive associations with SWB, while financial facility density (B = −0.035, *p* < 0.001) had a negative association. The primary determinants of SWB for elderly migrants ranged from individual to environmental factors. Through the enhancement of the accessibility to healthcare facilities in their new homes, in addition to promoting recreational activities and social services, the SWB amongst elderly migrants could be enhanced further.

## 1. Introduction

With the development of high-quality cities in China, the pursuit to improve people’s life and happiness has gradually replaced the simple yearning for wealth. Enhancing people’s subjective well-being (SWB) has become an important goal of the current government’s livelihood construction. SWB is an important psychological index of individual quality of life, and the term includes affective states, valuations of quality of life of different people, their residential environment, and the events they encounter [1]. The term SWB was first introduced by Edward Diener in 1984 as being central to a person’s experience, consisting of positive aspects, and a global assessment of a person’s life [2]. In 1995 he modified this definition by also including cognitive evaluations or appraisals of life satisfaction as a whole, and emotional reactions to life events [3]. In 2006 he developed the latest version, identifying SWB as a term for different valuations of people’s quality of life, the events that happen to them, their physical and mental health, and the circumstances in which they live [4]. Issues regarding the SWB of the elderly have been garnering more attention lately, as the rapid aging of the population has increasingly become a social challenge [5,6].

There are many factors that affect older adults’ SWB. However, the evidence regarding the influence of the factors of residential environment on the SWB of elderly migrants has been limited, unlike that regarding individual resources, and most studies have focused on the association between an individual’s attributes and SWB [7,8]. In fact, residential environment can have a substantial impact on elderly SWB, as they become less mobile due to deteriorating health status and because they tend to spend more time in their residential community [9,10,11]. Previous studies have confirmed that residential environment has a significant positive effect on SWB [8,12]. Liu et al., another study based in China, reported that residential environments have a stronger impact on SWB than do individual attributes [7]. The major environmental factors of SWB included residential environments of good quality with better socio-economic background in the neighborhood and ease of accessibility to basic facilities. The SWB of the older adults is highly influenced by the social and physical environments, as confirmed by several studies in this regard [7,8]. The physical aspect of a residential environment mainly focuses on the role of accessibility to local amenities, such as healthcare centers, banks, and parks, all of which are the locus of most material facilities related to older adults’ well-being [7,8,13]. For instance, good accessibility to green parks can provide an open, natural public space, which positively impacts SWB. The accessibility of a residence to the city center influences residents’ abilities to easily enjoy various services and improve residents’ SWB. The social aspect of the residential environment encompasses interpersonal relationships and neighborhood characteristics [14,15]. For instance, social cohesion, a measure of an individual’s social environment, can also influence individual health and SWB [8,16].

Internal migrants, people who travel a substantial distance but within the country, account for approximately 18% of the total population in China, and 7% of these internal migrants, or approximately 18 million people, are elderly [17]. The number of elderly migrants has grown rapidly in recent years due to the persistence of internal migration and aging trends in China [7,17]. Neglecting the elderly migrants having migrated to new cities, the majority of the earlier studies concentrated on the SWB of either the rural elders or the local elders in China [18,19]. Older adult internal migration is expected to be a universal phenomenon in China soon, which may affect the health of elderly migrants [5]. Policymakers should be aware of the situation, especially on the implications for elderly migrants’ SWB. However, it is only recently that evaluating elderly migrants’ SWB has received academic attention. These migrants were not provided the services and social benefits enjoyed by the local residents, as a result of the “hukou” restriction, a registry system of the households in China [5,20]. Impacting the well-being of the elderly migrants in a much higher manner as compared to the other local elders, the “hukou” system influenced inequality amongst the migrants [20,21]. Due to the environmental inequities and the difficulty of the migrants to adapt to the new cities (like interacting with neighbors), the elderly migrants of China were found by Gao et al. to display lower levels of SWB compared to the other local elderly (e.g., unequal access to healthcare facilities, social services, and resources) [20,22].

In addition to being interrelated and influencing each other, the social and physical environments also are hypothesized to influence SWB and health issues [23,24]. Hence, at both the community and the individual levels, it is imperative to measure residential environmental characteristics. Nevertheless, there has been very little effort towards the integration of these multi-level SWB factors, with the existing studies focusing on just one such level [18,24]. Applying a multilevel perspective, this study endeavors to fill in the knowledge gaps, assessing the factors that influenced the SWB of the elderly migrants. This study aimed to identify the determinants of subjective well-being of elderly migrants at the individual and environmental levels. The manner and the extent to which the SWB of elderly migrants is influenced by residential environments was the specific focus of this study. Our findings will provide useful information to help policy-makers devise suitable strategies to improve the SWB of elderly migrants in China by making appropriate changes to their residential environments.

## 2. Materials and Methods

### 2.1. Study Design and Setting

Between December 2018 and December 2019, a community-based questionnaire survey was conducted in Dongguan, an industrial city in the Guangdong–Hong Kong–Macau Greater Bay, in South China. The Dongguan area was identified for two specific reasons for this study. First, the largest migrant population of China resided in Dongguan. Second, regarding the cultural and its socio-demographic background, the migrant population of Dongguan was found to be heterogeneous in nature [25,26].

### 2.2. Participants

Participants included elderly migrants who had come at least six months prior to the study and were not listed in the Dongguan household registration system. This study defined an elderly person as aged 60 or more years. The eligible list of elderly migrants for this study was provided by the community committee. To identify the participants, a multi-stage cluster sampling survey technique was applied. In the first stage, 4 out of 33 districts were purposively selected (Songshan Lake, Dalang, Liaobu, and Dalingshan), each having more than 75 percent elderly in the population and all located within 30 km of Guangdong Medical University. In the second stage, 22 clusters, which included 20 migrant elders in each cluster, were randomly selected from 26 communities with a probability proportional to the elderly population density. After excluding 11 communities due to low population density, 15 communities were selected. In the third stage, within each cluster, migrant elderly participants were selected randomly. Finally, 470 elderly migrants participated in this study.

### 2.3. Measurements

#### 2.3.1. Subjective Well-Being (SWB)

SWB was assessed by the Memorial University of Newfoundland Scale of Happiness (MUNSH), which was designed specifically for older adults, and which has high validity (Kaiser–Meyer–Olkin of 0.703) and consistency (Cronbach’s alpha of 0.735). The MUNSH is a multi-item scale which has 24 items, with total scores ranging between −24 and +24 points, and where higher scores indicate better SWB [27].

#### 2.3.2. Residential Environment

Both the social and the physical environmental factors were measured. The walkability and the distances (< 1 km or ≥ 1 km) from the residence of the participant to healthcare facilities (such as hospitals, health centers, clinics, and nursing homes) and financial facilities (such as post office and banks) were included in the study of the physical environment. Google Maps^®^ was used to measure the distances from the participant’s home to each of the closest abovementioned facilities in kilometers. Participants were asked whether they had seen other people in their neighborhood walking around, if there was enough green space and walking area, and if there were sufficient opportunities or facilities for physical activities. Applying a scale Mujahid et al. developed, the walkability of the neighborhood was determined [28]. A 5-point Likert Scale, from 1 = strongly disagree to 5 = strongly agree with the statements, was applied in the questionnaire; 0.73 was the original scale’s Cronbach alpha, with the total score ranged between 7 and 35, where higher scores indicated better walkability [28].

Social environment included distances (< 1 km or ≥ 1 km) from home to the closest culture facilities (cultural centers, libraries) and recreation facilities (elderly activity centers, parks, and cinemas) and social cohesion. Google Maps^®^ was again used to measure the distances from the participants’ homes to each of the closest abovementioned facilities in kilometers. Asking the respondents four questions, including their relationship with their neighbors and their values such as their inter-personal trust, the good relationship between members of the community, also known as social cohesion, was determined by the Neighborhood Relation Scale developed by Mujahid et al. [22,28]. The 5-point Likert Scale, having responses between 1 = strongly disagree to 5 = strongly agree with each statement, was applied in the questionnaire. With the total score ranging between 4 and 20, the Cronbach’s alpha of the original scale was found to be 0.74, where higher scores indicated better social cohesion [28].

The numbers and details of the recreational, cultural, health, and the financial facilities in every community were also compiled. The number of recreational/cultural/health/financial facilities divided by the total area in square kilometers of each community determined the density of the recreational/cultural/health/financial facilities.

#### 2.3.3. Individual Characteristics

We measured individual characteristics including demographic information and health condition. The demographic characteristics recorded were gender, age, and marital status (married or cohabiting vs. other). Details of health conditions such as depressive symptoms and self-rated health were also compiled. Self-rated health was divided into 3 ordinal categories: “good”, “fair”, and “poor”. Containing twenty questions as per the recommendations of the World Health Organization to screen depression, the Zung Self-rating Depression Scale was used to measure the depressive symptoms of the participants, with scores ranging between 20 and 80. A score higher than 53 indicated possible clinical depression [29].

### 2.4. Data Collection

Nine interviewers were trained by an experienced researcher before formally collecting data. The questionnaires were tested in a pilot study. The structured questionnaire as mentioned above was used to conduct the personal interviews of all the participants using their local language at their homes, with each interview lasting around 20–25 min. The administrative committees from the communities in which the participants resided provided the information regarding the community.

### 2.5. Statistical Analysis

For quantifying the effects of the community environment and the individual attributes, the multi-level linear models were found suitable. To account for clustering at the community level, within the 15 communities (level 2), the individuals (level 1) were nested. Considering only the clustering by community, the null model (no covariates) was applied for an estimation of the intercept and residual variance. The following were the equations for the null model:

Level 1 model (individual level): *Y_ij_* = *β*_0*j*_
*+ r_ij_*

Level 2 model (communities): *β*_0*j*_ = *γ*_00_
*+ μ*_0*j*_

Combined model (fixed model): *Y_ij_* = *γ*_00_
*+ μ*_0*j*_*+ r_ij_*

where *Y_ij_* is the SWB of the *i*th person in the *j*th community, and where *r_ij_*_=_ random error associated with *i*th person in the *j*th community. *β*_0*j*_ is the community-specific (random) intercept, where *γ*_00_ = the overall mean SWB across all communities; *μ*_0*j*_ = a series of random deviations from the mean SWB or QoL for the community.

The relationship between the community-level and individual-level attributes of communities with SWB was examined subsequent to the testing of the community-level variance in the SWB excluding all explanatory variables (Model 1 and Model 2, respectively). Then, we modeled all individual- and community-level variables simultaneously (Model 3). Two-level linear regression analyses with random intercept models were fitted using the −2 log likelihood (−2 LL) and Akaike information criterion (AIC) for model selection. All analyses were done using R version 3.4.2.

## 3. Results

There was a total of 470 migrant elderly, and the mean age was 67.5 ± 5.5 years. The ratio of males (47.9%) to females (52.1%) was approximately 1:1, and most were married or cohabitating (79.8%). Most said they had fair to good health (90.2%). Generally, elderly migrants lived within 3 km of facilities that provided basic needs such as financial and healthcare facilities as well as cultural and recreational facilities. However, the migrants lived closer to financial and health facilities but further from cultural and recreational facilities. They had good walkable environments (median score = 28) and social cohesion (median score = 16). The community-level residential environment determinants, namely, density of each facility, are also shown in Table 1.

At both the community and individual levels, to assess the determinants of SWB, multi-level models were applied. The regression models were expanded further to examine community-level factors influencing elderly SWB using linear mixed models. The null model indicated that there was a statistically significant variation in SWB across communities (*X*^2^ = 167.23, *p* < 0.05). A variance of SWB of 26.1% was indicated by the random effect across communities, with the intra-class correlation coefficient (ICC) being 0.261.

The results of the determining factors and the multi-level analysis on the SWB of the elderly migrants are displayed in Table 2. Model 1 included all the variables at the individual level. After adjusting the various covariates, the strongest association with SWB was found in the case of depression. Compared to those who were depressed, individuals who were not depressed indicated a higher SWB by 0.732 points. Higher SWB was found to be significantly related to good self-rated health conditions, namely, married/cohabiting, and old age. Nevertheless, social cohesion, walkable environment, and closeness to recreational facilities were weakly associated with SWB. There was inclusion of only the community level variables in Model 2. Whereas the density of financial facilities displayed a negative association (B = −0.044, *p* < 0.01), the densities of recreation facilities (B = 0.038, *p* < 0.01) and of health facilities (B = 0.035, *p* < 0.01) indicated a positive association with SWB. Both the community- and individual-level variables were included in Model 3 simultaneously. Significant associations with SWB were illustrated by age, marital status, self-rated health, depression, distance to closest recreation facility, social cohesion, and density of health facility, and financial facility and recreation facility remained significantly associated with SWB when the other covariates were controlled, except for walkable environment (B = 0.014, *p* > 0.05). That this model was capable of explaining better the variation in SWB, compared to the other two models, was implied by Model 3 indicating a much smaller AIC value.

## 4. Discussion

This study assessed the associated factors of SWB of elderly migrants in Dongguan city, one of the most rapidly developing areas of China, using both single-level and multi-level models. SWB is associated with various factors, ranging from individual to environmental factors. The results shed light on multi-disciplinary and multi-level factors of SWB, particularly focusing on the extent to which, and the ways in which, migrant elderly people’s residential environments influence their SWB.

This study found that physical and social environments affect the SWB of elderly migrants. However, environmental factors exert a weaker effect on older adults’ SWB than individual factors, which is consistent with the quantitative results from the same migrant elderly cohorts [5], but different from the result from older adults in Shanghai [7]. Health conditions highly contribute to their SWB, and depression has the strongest association with SWB. In addition to improving their lives, good health was found to be the most significant prerequisite for SWB in elders, as indicated by earlier studies [5,30]. Older adults were more likely to report higher levels of SWB when they had a positive attitude for aging [31]. The study by Kosloski et al. found that depression was associated with lower SWB in older adults, especially among elderly migrants who were acclimatizing to a new community [32].

We found that living near recreation facilities and having good social cohesion are associated with high SWB for elderly migrants. These findings are consistent with Lin’s results that an increase in opportunities for leisure activities with other people leads to positive SWB [33]. Moreover, the neighborhood in which the elders lived had an important bearing on their SWB, as indicated by the results. Previous studies verified that a cohesive neighborhood offers migrants support and alleviates their life stress [33,34]. Our study also showed that the density of health facilities is positively associated with good SWB, while the density of financial facilities negatively impacts SWB. Thus, the importance of healthcare to the elderly migrants and their effects on SWB were implied. This could be related to the idea that health status is strongest factor of SWB among older adults. Good accessibility to health facilities has a strong effect on SWB, while this outcome is in consonance with that of the existing literature [7,8]. The overcrowding and noise in areas with a high density of financial facilities explained the negative influence on the SWB of elderly persons by the density of the financial facilities [35,36]. Most elderly migrants in this study lived in the city center, which has more financial facilities and transportation. Thus, the financial facility-dense areas where migrants live have more noise and industrial pollution. This result is different from that of a study on older adults in Shanghai, which showed that good accessibility to financial facilities seemed to positively influence older adults’ perceptions of personal relationships and affection [7].

Certain limitations of this study need to be noted. First, temporal ambiguity (a concept where researchers cannot determine whether an exposure preceded an outcome) concerning causes and effects may be present. Second, memory recall could have been an issue, since the information obtained from each participant was self-reported. Third, the effects may not have been interpreted causally, since in the multi-level analysis, contextual effects tend to be interpreted by the individual averages used as group effects.

Based on the findings discussed above, the following policy implications are recommended. First, in addition to providing suitable community-based support, the social services policies need to look into the needs of the elderly migrants. Second, the following planning strategies are needed: enhancing physical planning approaches (e.g., the public space of communities), and promoting access to healthcare by planning the service delivery for the whole community. Third, for promoting mutual help and the interaction between the local elderly population and the migrants, community events, recreational spaces, and healthcare centers would be indispensable.

## 5. Conclusions

This study assessed the determinant of SWB of the migrant elderly, integrating individual, community, and environmental factors. The study included both individual- and community-level variables using a multi-level sampling frame and analysis. By enhancing their accessibility to healthcare and recreational activities, and promoting social services in their new homes, the SWB amongst elderly migrants could be enhanced further. To ensure that the elderly do not undergo undue mental health problems, this study suggests further evaluation periodically based on how the evidence evolves.

## Figures and Tables

**Table 1:** Summary of participants’ characteristics and residential environment of individual-level and community-level.

Variable	N	%	Median	Range
Age (years)				
60–69	333	70.9		
70–79	123	26.2		
80–99	14	3.0		
Gender				
Male	225	47.9		
Female	245	52.1		
Marital status				
Married/cohabitating	375	79.8		
Other	95	20.2		
Self-rated health				
Good	211	44.9		
Fair	213	45.3		
Poor	46	9.8		
Depression				
Yes	99	21.1		
No	371	78.9		
Distance to closest financial facility (km)	470		0.50	0.13–4.09
Distance to closest health facility (km)	470		1.00	0.15–3.27
Distance to closest cultural facility (km)	470		2.00	0.12–15.12
Distance to closest recreation facility (km)	470		2.00	0.18–9.76
Walkability (score)	470		28.00	7.00–35.00
Social cohesion (score)	470		16.00	4.00–20.00
Health facility density (facilities/km^2^)			4.95	0.88–19.02
Financial facility density (facilities/km^2^)			5.03	0.65–12.23
Culture facility density (facilities/km^2^)			1.72	0.23–5.26
Recreation facility density (facilities/km^2^)			2.99	0.43–7.65

**Table 2:** Individual and community factors of participants’ subjective well-being.

	Model 1		Model 2		Model 3	
	Estimate	SE	Estimate	SE	Estimate	SE
**Fixed part**						
***Individual-level variables***						
Gender (ref. = male)						
Female	0.157	0.083			0.079	0.096
Age (ref. = 60–69 years)						
70–79 years	0.087	0.049			0.052	0.057
80–99 years	0.107 *	0.084			0.096 *	0.042
Marital status (ref. = other)						
Married/cohabitating	0.203 *	0.078			0.185 *	0.078
Self-rated health (ref. = poor)						
Good/fair	0.248 *	0.092			0.236 *	0.093
Depression (ref. = yes)						
No	0.732 ***	0.079			0.745 ***	0.079
Distance to closest financial facility (ref. ≥ 1 km) <1 km	0.159	0.082			0.146	0.085
Distance to closest health facility (ref. ≥ 1 km) <1 km	0.201	0.074			0.198	0.075
Distance to closest culture facility (ref. ≥ 1 km) <1 km	0.195	0.082			0.146	0.085
Distance to closest recreation facility (ref. ≥ 1 km) <1 km	0.239 ***	0.066			0.227 ***	0.067
Walkability (ref. < 28)						
≥28	0.018 *	0.006			0.014	0.007
Social cohesion (ref. < 16)						
≥16	0.077 ***	0.013			0.079 ***	0.014
***Community-level variables***						
Health facility density			0.035 **	0.013	0.026 *	0.014
Financial facility density			−0.044 **	0.014	−0.035 **	0.012
Culture facility density			0.017	0.029	0.015	0.030
Recreation facility density			0.038 **	0.012	0.032 *	0.013
**Random effect**						
Community-level variance	0.176	0.135	0.291	0.155	0.204	0.123
Model fit						
−2 LL	2355.3		2659.8		2281.3	
AIC	2329.3		2671.8		2227.3	

ICC = 0.261; * *p* < 0.05; ** *p* < 0.01; *** *p* < 0.001.

## Data Availability

Data are available upon request from the corresponding author.
